# Less is more: slow-codon windows enhance eGFP mRNA resilience against RNA interference

**DOI:** 10.1098/rsif.2024.0582

**Published:** 2025-03-19

**Authors:** Judith A. Müller, Gerlinde Schwake, Anita Reiser, Daniel Woschée, Zahra Alirezaeizanjani, Joachim O. Rädler, Sophia Rudorf

**Affiliations:** ^1^ Ludwig-Maximilians-Universität, Faculty of Physics, Munich 80539, Germany; ^2^ Independent Researcher, Wuppertal, Germany; ^3^ Leibniz University Hannover, Institute of Cell Biology and Biophysics, Hannover 30419, Germany

**Keywords:** slow-codon windows, mRNA, RNA interference, mRNA stability, single-cell

## Abstract

Extensive efforts have been devoted to enhancing the translation efficiency of mRNA delivered to mammalian cells via codon optimization. However, the impact of codon choice on mRNA stability remains underexplored. In this study, we investigated the influence of codon usage on mRNA degradation kinetics in cultured human cell lines using live-cell imaging on single-cell arrays. By measuring mRNA lifetimes at the single-cell level for synthetic mRNA constructs, we confirmed that mRNAs containing slowly translated codon windows have shorter lifetimes. Unexpectedly, these mRNAs did not exhibit decreased stability in the presence of small interfering RNA (siRNA) compared with the unmutated sequence, suggesting an interference of different concurrent degradation mechanisms. We employed stochastic simulations to predict ribosome density along the open reading frame, revealing that the ribosome densities correlated with mRNA stability in a cell-type- and codon-position-specific manner. In summary, our results suggest that the effect of codon choice and its influence on mRNA lifetime is context-dependent with respect to cell type, codon position and RNA interference.

## 1. Introduction

Regulating mRNA stability holds significant importance not only for clinical applications [1] but also within the realm of synthetic biology. Factors that compromise mRNA stability provide potential targets for stability engineering. In this context, the choice of mRNA sequence is particularly important, specifically the mRNA 5′-cap [2], nucleotide modifications [3], untranslated region (UTR) design [4,5], poly(A) tail length [6–8], RNA interference (RNAi) binding sites [9] as well as codon usage [10,11]. Understanding the underlying mechanisms of these factors is crucial to achieving improved control over the half-life of mRNAs.

Degeneracy of the genetic code enables encoding of the same amino acids via multiple synonymous codons [12–14]. These synonymous codons are recognized by specific transfer RNAs (tRNAs), and the decoding rate of each one is distinct. The choice of codons determines the translation process and the folding dynamics of the nascent peptide chain [15]. In nature, genetic codes exhibit codon bias, with diverse organisms displaying varying frequencies of synonymous codons. Optimal codons are those synonymous codons that can be translated more rapidly and accurately [16,17]. Codon optimality influences translation in several ways, i.e. by changing ribosome speed [18–23], translation efficiency [24–26], protein folding [21,27,28] and translation fidelity [15,16]. Various computer models have been developed to predict efficient synonymous codon exchange [20,29–34]. A comprehensive review can be found in Hanson & Coller [15]. Sequence optimization is often performed aiming for enhanced translation [30,35,36]. Recently, Trösemeier *et al*. introduced software for stochastic simulations of mRNA translation [37]. The software, named OCTOPOS, takes into account many parameters relevant for the translation process to optimize or de-optimize mRNA sequences. To this end, a machine-learning method is applied to combine the simulation results with further mRNA-specific features (such as abundance or length) into a comprehensive model for protein output prediction. In addition to generating optimized mRNA sequences, OCTOPOS calculates steady-state ribosome density profiles, i.e. the ribosome occupancy of individual codons of a sequence

**Figure 1.**
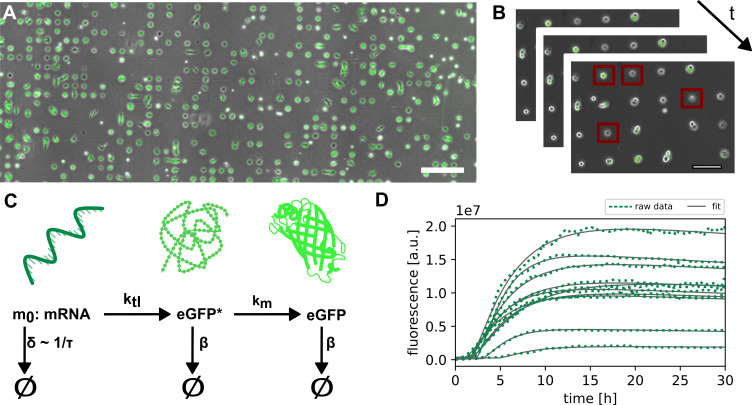
Platform for LISCA. (*A*) A549 cells on single-cell array 24 h after transfection. Bright-field and eGFP-fluorescence overlay. Scale bar corresponds to 400 μm. (*B*) Illustration of the time resolution in single-cell translation experiments with the application of a region of interest (indicated with red squares). (*C*) Three-stage model for mRNA translation and protein maturation with mRNA stability *τ* (inverse of mRNA degradation rate *δ*), translation rate *k*_tl_, maturation rate *k*_m_ and protein degradation rate *β*. (*D*) Exemplary eGFP fluorescence trajectories of single cells (green) recorded over 30 h, fitted to the three-stage translation and maturation model (grey).

The effect of ribosome density on translation as well as mRNA stability is not yet fully understood. An inverse correlation between ribosome movement or density along the open reading frame (ORF) and mRNA stability has been observed [38–41] and shown to play a critical role in regulating gene expression [42,43], and potential ribosome stalling sequences are targeted via no-go decay [44–50]. In contrast, experimental studies indicated that mRNA covered by ribosomes exhibits protection against decay [51–55]. Deneke *et al*. [56] developed a theoretical model that links mRNA degradation and translation based on the assumption that ribosomes protect the mRNA against endonucleolytic degradation processes. Ruijtenberg *et al.* discovered that translating ribosomes play a role in unmasking mRNA, thereby making it accessible for target recognition in RNAi-mediated mRNA cleavage [57]. Resolving impacts on mRNA stability based on modifications in predicted ribosome occupancy requires precise measurement of translation and degradation kinetics [58]. However, disentangling mRNA translation and stability in the context of codon usage is experimentally challenging. In previous work, it was shown that enhanced green fluorescent protein (eGFP) reporter translation and lifetime are simultaneously measured using live-cell imaging on single-cell arrays (LISCA) [59–61]. LISCA monitors the time courses of mRNA-mediated eGFP fluorescence in hundreds of individual cells on a micro-pattern in parallel. From the dynamics, both mRNA translation and degradation rates are determined based on a kinetic reaction model for mRNA translation including protein maturation as well as protein and mRNA degradation (figure 1C) [60,62]. In previous work, Ferizi *et al.* used the LISCA technique to optimize the UTR region of therapeutic mRNA sequences resulting in improved functional mRNA lifetime [4].

Here, we systematically study how slow-codon sequences modulate the stability of mRNA constructs with defined small interfering RNA (siRNA) target sites. To achieve this, we designed five constructs of eGFP-mRNA with slowly translated codon windows at different positions within the ORF. We used OCTOPOS to simulate the translation of these constructs and predict their ribosome density profiles. To assess intracellular stability, we employed LISCA to measure the translation and degradation kinetics of the designed mRNA constructs across hundreds of transfected cells in parallel. Fitting the data to a translational rate model, we calculated degradation and expression rates independently. First, we quantified the decrease in stability of the original eGFP-mRNA construct in the presence of siRNA targeting one of two selected siRNA-binding sites. Exact quantification was achieved through co-transfection and normalization with a Cayenne red fluorescence protein (CayRFP)-mRNA control. Next, we examined whether siRNA-mediated knockdown is influenced by synonymous codon changes. Correlating the simulated ribosome density profiles with the measured mRNA lifetimes revealed a distinct correlation profile between measured stabilities and predicted ribosome density. We show that the presence or absence of RNAi changes the effect of codon choice on mRNA lifetime.

## 2. Results

### 2.1. Live-cell imaging on single-cell arrays

We assess mRNA stability from quantitative analysis of single-cell eGFP gene expression time courses with high temporal resolution. To this end, we acquire hundreds of individual single-cell fluorescence trajectories using the LISCA approach. As in previous work [60,62], human lung carcinoma A549 or human liver carcinoma HuH7 cells were seeded in microscopy cell-culture channel slides with micro-patterned surfaces consisting of 10 000 cell-adhesive squares (20 × 20 µm) allowing for attachment of one cell per spot (figure 1A). Micro-fabrication was carried out using a refined method (see §4 and electronic supplementary material for details). After an incubation time of 2 h, cells were transfected with reporter mRNAs. Depending on the experimental approach, the mRNA transfection was followed by siRNA transfection. Long-term scanning time-lapse bright-field and fluorescence imaging was carried out under physiologic conditions in order to follow eGFP and CayRFP translation dynamics (figure 1B). Subsequently, fluorescence time traces of individual cells were extracted from the image time series using Python-based in-house cell tracking software (Python-based automated microscopy analysis (PyAMA)) [63,64] (figure 1D). Fluorescence time courses were fitted to a kinetic model of protein expression including mRNA translation, mRNA degradation, protein maturation and protein degradation as illustrated in figure 1C,D. The analysis yields individual values for the kinetic rates per cell for mRNA degradation, *δ*, and the initial expression rate *m*_*0*_ *× k*_tl_, where *m*_*0*_ denotes the number of mRNA molecules translated and *k*_tl_ the translation rate (electronic supplementary material, figure S1). Protein maturation and degradation rates were analysed in previous work and fixed as described earlier [60]. This allowed precise fitting of each fluorescence trace as illustrated in electronic supplementary material, figure S2. In the following results, we discuss mRNA stability (or lifetime), *τ*, as the inverse of the measured mRNA degradation rate *δ*. In each assay, cells were co-transfected with the mRNA of interest (i.e. one of the eGFP mRNA variants) together with a reference reporter mRNA (CayRFP). Co-transfection analysis of eGFP and CayRFP fluorescence results in a distinct eGFP and CayRFP fluorescence trajectory for every single cell. This then allows us to normalize the eGFP mRNA parameters with the respective CayRFP mRNA parameters of the same cell as explained in electronic supplementary material, figure S3. Thereby, we address intrinsic cell-to-cell variance in expression levels caused by varying numbers of lipoplex particles delivered per cell as well as varying cell cycle or metabolic state of each cell. All kinetic rates are reported in terms of fold change of eGFP mRNA kinetic rates normalized to the CayRFP mRNA reference value (electronic supplementary, figures S3 and S4). mRNAs were pre-mixed before lipoplex formation in order to secure equal amounts of mRNA molecules per lipoplex [65]. As previously reported, the two-colour single-cell referencing approach enhances the accuracy of measurement of mRNA degradation and expression rates yielding an improved signal-to-noise ratio up to a factor 5 compared with population measurements [66]. As control, single-cell correlation analysis was performed. No correlations were found in eGFP and CayRFP mRNA degradation rates, while some correlations were observed in initial eGFP and CayRFP mRNA translation rates *m*_*0*_ *× k*_tl_ (electronic supplementary material S5). The latter are the result of eGFP/RFP mRNA co-delivery and the dominating influence of lipoplex number fluctuations that determine the amount of initial number *m*_*0*_ of mRNA released.

### 2.2. Quantification of RNAi-mediated mRNA decay

**Figure 2.**
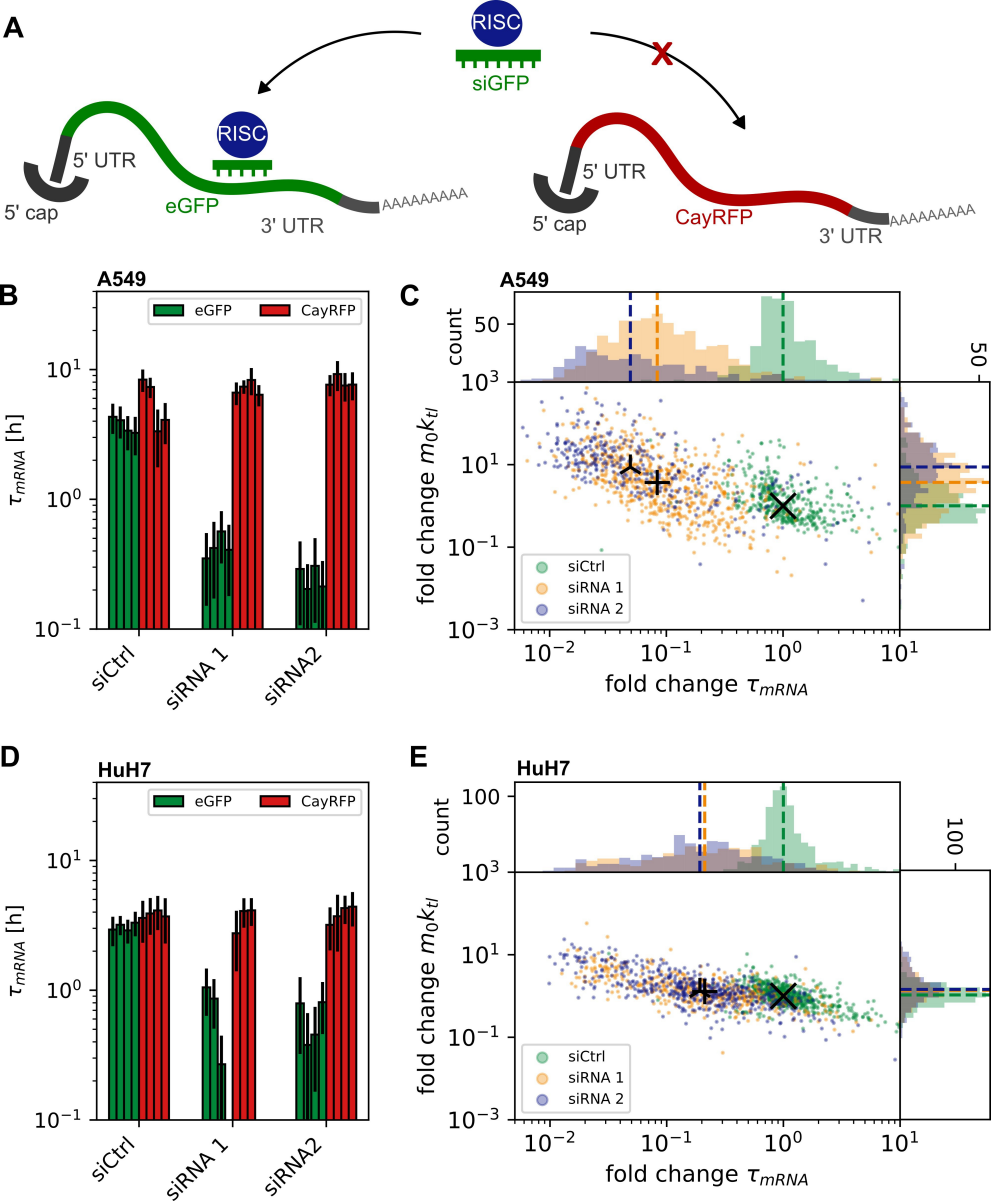
Determination of mRNA stability at the single-cell level: (*A*) Specific RNAi-mediated degradation by siRNA targeted to the ORF of eGFP but not CayRFP mRNA. (*B, D*) Single-cell mRNA median half-life (bars) with median absolute deviation (error bars), demonstrating the decrease in eGFP mRNA stability in the presence of siRNA 1 or 2 but not siCtrl (non-binding control), with no impact on the stability of co-transfected CayRFP mRNA. Shown are medians of single cells for separate experiments. (*C, E*) Scatter plots and corresponding histograms show decrease in median mRNA stability but constant initial mRNA expression rate compared with the median of experiments with siCtrl. Individual single-cell data of each experiment were normalized to respective CayRFP mRNA values for each cell and then normalized to aggregated siCtrl data. (*B*) and (*C*) show data measured in A459 cells, (D) and (E) respective data for HuH7 cells.

Specific and targeted triggering of mRNA degradation using siRNA allows for quantification of RNAi-mediated mRNA decay as depicted schematically in figure 2A. We performed LISCA on cells transfected with siRNA in addition to eGFP- and RFP-mRNA. The siRNA was targeted against the ORF of eGFP mRNA with binding sites at nucleotide position 122 (siRNA 1) and 433 (siRNA 2), respectively; see electronic supplementary material for details. A non-binding siRNA (siCtrl) was used as reference control. To confirm the specificity of eGFP-mRNA targeting over RFP mRNA, we assessed their stabilities in the presence and absence of siRNA. Figure 2B–D shows siRNA-induced knockdown in terms of eGFP-mRNA lifetime reduction. The lifetime of eGFP mRNA is reduced in the presence of both siRNAs compared with siRNA control (figure 2B,C), with a median fold change of normalized eGFP mRNA stability of 0.08 ± 0.05 for siRNA 1 and 0.05 ± 0.03 for siRNA 2 in A549 cells. In HuH7 cells, the difference was found to be 0.21 ± 0.16 for siRNA 1, and transfection of siRNA 2 led to a fold change of 0.21 ± 0.13. In contrast to the eGFP mRNA stability, the CayRFP reference construct’s stability remains unaffected with siRNA 1 or siRNA 2 (see figure 2B,D). Notably, the normalized initial expression rates *m*_*0*_ *× k*_tl_ are not influenced by the presence of eGFP mRNA-specific siRNA in HuH7 cells and only slightly influenced in A549 cells (see figure 2C,E) and no correlation exists between expression rates and mRNA stability at the single-cell level (figure 2C,E and electronic supplementary material, figure S1), or between eGFP- and RFP-mRNA (electronic supplementary material, figure S5). These results demonstrate the capability of LISCA to quantitatively measure mRNA stability independently of translational speed and that RNAi can serve as a suitable tool to study mRNA stability in LISCA experiments.

### 2.3. Generation of predicted slow-codon windows

**Figure 3.**
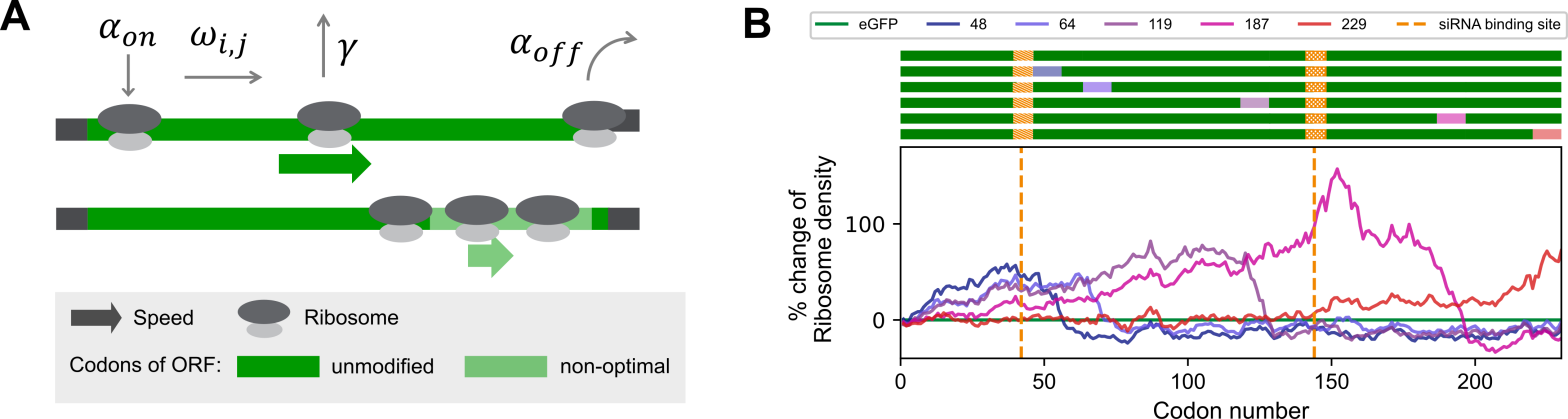
Computational predictions of ribosome density modulations on the ORF of eGFP mRNA. (*A*) Scheme of the modelling concept with binding rate *α*_on_, elongation rates *ω*_*i,j*_ of codons *i* in mRNA *j*, premature drop-off rate *γ* and translation termination rate *α*_off_. Replacement of stretches of original codons (dark green) by their synonymous but non-optimal alternatives (light green) is assumed to cause a slowdown of translation at these slow-codon windows, which can lead to a local increase in ribosome density. (*B*) Top: ORF of eGFP mRNA variants (dark green) with slow-codon windows (coloured boxes at indicated positions) and siRNA-binding sites (orange boxes). Bottom: Predicted differences of ribosome density profiles along the ORF of unmutated eGFP (green) and variants with slow-codon windows at indicated positions (purple to red). Numbers indicate the position of the first inserted slow codon. Ribosome density was predicted to increase both upstream and at the site of the inserted slow-codon window but to decrease further downstream to levels below those predicted for the unmutated control. These simulations were performed using the previously published OCTOPOS software [37,70,71].

Next, we study the influence of non-optimal codons in the ORF on mRNA half-life in the context of RNAi. Prior to target mRNA cleavage, a siRNA/RNA-induced silencing complex (RISC) needs to bind to the target mRNA and find the cleavage site. Ribosomes and the translational machinery are known to interfere with this process [57,67–69]. Here, we aimed to modulate ribosome density in a site-specific manner and investigate the effect on mRNA stability under siRNA attack. This was achieved by synonymous codon exchange. Depending on their optimality or de-optimality, codons are translated at different rates, leading to modulations of ribosomal speed along the ORF (figure 3A) [11,72,73]. A stretch of several consecutive non-optimal, slow codons was described to cause a local increase in ribosome density, especially if those codons are preceded by codons with higher optimality [74]. Due to the stochastic nature of translation elongation, ribosome jams or collisions might occur [38,45,47,74]. Thus, one way to control translation and thereby ribosome speed on the ORF is to replace individual codons with synonymous but more slowly translated non-optimal codons. We simulated 230 synonymous variants of eGFP mRNA, each containing a window of 10 adjacent non-optimal but synonymous codons at a different position. Using our software OCTOPOS [37,70,71,75], we simulated ribosome movement on the ORF of these 230 variants and identified five sequences with high similarity in terms of overall ribosome flux (electronic supplementary material, figure S6). Predicted ribosome densities along the ORFs of these five constructs compared with unmutated eGFP mRNA are shown in figure 3B. The simulations show an increased ribosome density for all constructs with the peak occupancy in proximity of the non-optimal codon window. Changing the translation initiation rate in the simulations within physiological limits alters the shape of the resulting ribosome density profiles but not the overall effect (electronic supplementary material, figure S7).

### 2.4. mRNAs with non-optimal codon windows show decreased stability

**Figure 4.**
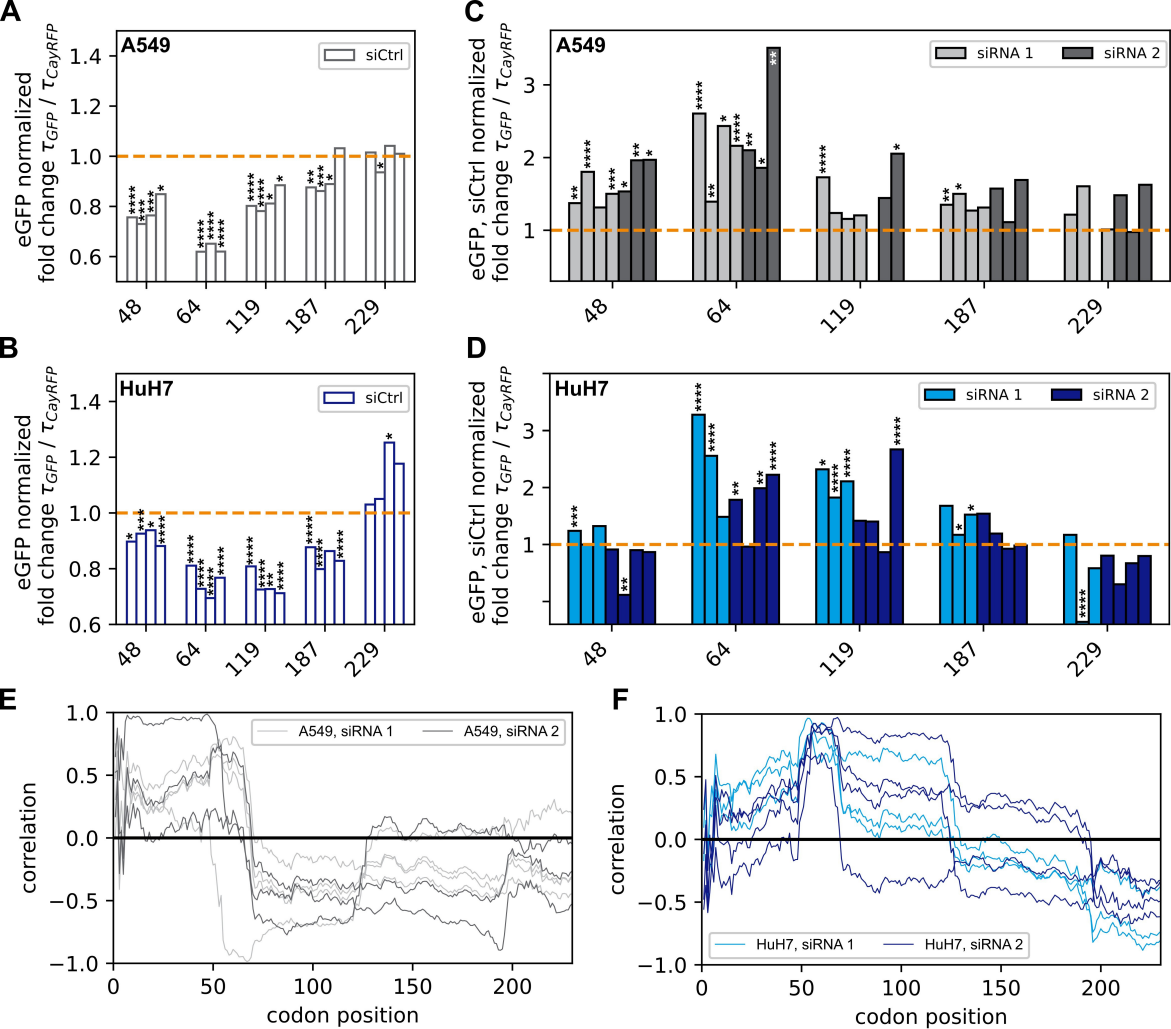
Destabilization versus stabilization—effect of non-optimal codons on mRNA stability: the stability of eGFP mRNA was assessed using LISCA and normalized to equivalent values from reference CayRFP mRNA for each cell. (*A*) In control experiments, constructs with non-optimal codon windows exhibited a decreased median stability compared with unmodified eGFP mRNA in A549 cells. (*B*) Similar results were observed in HuH7 cells. (*C*) Upon addition of siRNA 1 or 2, the median stability relative to eGFP was restored compared with control experiments with unmutated eGFP. Bars show independent experiments. Data represent median single-cell values normalized to internal CayRFP control and siCtrl experiment. Statistical significance was determined using the Mann–Whitney U-test. Detailed single-cell data are provided in the electronic supplementary information. (*D*) Equivalent data collected in HuH7 cells. (*E*) Correlation of measured mRNA stability with simulated ribosome density in A549 cells is largely independent of siRNA-binding site. (*F*) The HuH7 data show similar trends to A549 cells, approximately up to position 70 and beyond position 130, with notable differences in the intervening region. The *p*-values correspond to results from Mann–Whitney *U* tests and are indicated in the plots as assigned in the following: **** for *p* < 10^–4^, *** for *p* < 10^–3^, ** for *p* < 10^–2^, * for *p* < 5 × 10^–2^ and ns for *p* > 5 × 10^–2^

It was described previously that—in absence of any siRNA—locally increased ribosome densities can potentially trigger cellular quality control mechanisms leading to mRNA degradation [11,22,74]. To test this within our single-cell experimental approach, we transfected A549 and HuH7 cells, respectively, on a single-cell array with mRNA encoding either eGFP or one of the five synonymous variants featuring slow-codon windows, as outlined in the previous section. CayRFP as internal reference was co-transfected. By fitting the three-stage maturation model to the experimentally determined single-cell fluorescence trajectories, we assessed the stability of the variant mRNAs. We standardized these results using the co-transfected reference CayRFP mRNA and normalized them to the corrected stability of the unmutated eGFP mRNA, as explained earlier. All constructs exhibited either similar or decreased stabilities compared with unmutated eGFP mRNA (figure 4A,C and electronic supplementary material, figure S8 for single-cell data). Mann–Whitney *U* tests revealed significant changes for all constructs except for the mRNA with a slow-codon window positioned near the end of the ORF (codon positions 229–239) in the A549 cell line (figure 4A). A similar, although not identical trend, was observed in the HuH7 cell line. Here, we also observed mostly impaired stabilities (see figure 4C). The most substantial impact on mRNA stability, reducing it with a factor of 0.66 ± 0.07 compared with unmutated eGFP mRNA stability, was observed for the mRNA with a slow-codon window from position 64–74 in the A549 cells. The strongest reduction in HuH7 cells was observed for the slow-codon window starting from position 119, with a relative stability of 0.71 ± 0.06. It is noteworthy that the initial expression rates *m*_*0*_ *× k*_TL_ also varied compared with unmutated eGFP mRNA but with no discernible trend towards overall increased or decreased expression in either of the tested cell lines, as illustrated in electronic supplementary material, figure S9*A*,*C* (electronic supplementary material, figure S8 for single-cell data).

### 2.5. siRNA-mediated degradation mitigates the stability disadvantage of mRNAs with non-optimal codon windows

To test the effect of slow-codon windows on the effectiveness of RNAi-mediated degradation, we transfected A549 and HuH7 cells with CayRFP and either unmutated eGFP mRNA or one of the slow-codon window variants, followed by transfection with either negative control siRNA (siCtrl), or targeting siRNA variant 1 or 2. We determined the stability of the transfected mRNAs for each cell by LISCA, where we normalized both to the stability of the transfection control CayRFP mRNA and of the unmutated eGFP mRNA experiment; see electronic supplementary material, figure S8, for single-cell data. As expected, siCtrl did not have a systematic effect on mRNA stability compared with transfection in the absence of additional siRNA, and we obtained similar results as in the absence of any siRNA, i.e. constructs with slow-codon windows are less or equally stable than unmutated eGFP mRNA (figure 4A,C). Additionally, we computed the correlation coefficient between the measured stabilities and the simulated ribosome densities for each codon position within the ORF. This analysis revealed consistent correlation profiles for both cell lines when siCtrl was transfected. Specifically, we observed a strong negative correlation between simulated ribosome density and mRNA stability at the 5′ end of the ORF, which transitions to a positive correlation towards the 3′ end (electronic supplementary material, figure S12). These results suggest that higher ribosome occupancy at the beginning of the ORF negatively impacts mRNA stability, whereas higher ribosome density at the end of the ORF is beneficial. Introducing siRNAs targeting the eGFP mRNA constructs leads to an overall reduction of stability for all tested constructs (electronic supplementary material, figure S10). When comparing the fold-change stabilities of the mutated constructs with unmutated eGFP mRNA, we observe that the constructs with slow-codon windows are now as stable or more stable. Importantly, unlike the scenario without functional siRNA, where we observed a significant decrease in stability, we now mostly observe an increase in mRNA stability compared with the unmutated eGFP sequence and normalized to the control siRNA data (figure 4A–D). This indicates that the stability of mutated and unmutated constructs is at least equal under RNAi conditions, and for individual cases, we even find a significant stabilization. This means that RNAi diminishes the stability of mRNAs containing slow-codon windows by a smaller factor compared with unmutated eGFP mRNA. This change in stability is subtle compared with the overall effect of RNAi, underscoring the importance of a sensitive experimental approach.

### 2.6. mRNAs with non-optimal codon windows are less affected by siRNA-mediated degradation

To better assess the relative stabilization of mRNAs under RNAi by slow-codon windows, we further normalize mRNA lifetimes measured in the presence of siRNA 1 or 2 by the respective median values obtained in the presence of non-functional siCtrl. By doing so, it is possible to examine the relative influence of RNAi on mRNA stability in isolation regardless of other effects that might influence the stability such as quality control pathways or transfection-associated effects. We found a relative stabilization of up to 2.2 ± 0.4 fold in A549 cells and up to 1.4 ± 0.2 fold in HuH7 cells (see figure 4C,D). However, the normalized mRNA lifetimes correlate only partially with simulated ribosome densities at the specific siRNA-binding sites (see electronic supplementary material, figure S11). Notably, we also observed increased relative stabilities of mRNAs with non-optimal codon windows positioned upstream of the binding site of siRNA 2, which were predicted to not have increased ribosome densities at the siRNA-binding site in our simulation. Therefore, we again determined the coefficient of correlation of the strength of stabilization and ribosome density for each codon position along the ORF (see figure 4E,F and electronic supplementary material, figure S12). Here, we found that higher ribosome occupancy provides greater protection against siRNA-mediated degradation within the first 70 codons in A549 cells and similarly for the major part of the ORF in HuH7 cells. Conversely, towards the 3′ end, the simulated ribosome densities show an anti-correlation with experimentally determined mRNA stability.

In summary, our findings indicate that under conditions where mRNA is subjected to siRNA/RISC interference, non-optimal codon windows exhibit a protective effect, despite causing a reduction in stability in the absence of functional siRNA. For the two examined siRNAs, this protective effect is largely independent of the targeted cleavage site but seems to depend on the position of the non-optimal codon window and to be positively correlated with the ribosome density in the first part of the ORF, with some cell-type specific variations. The combination of LISCA and stochastic simulation makes the signature of position-dependent correlations accessible, providing interesting new approaches to studying the molecular details of RNAi.

## 3. Discussion

In this study, our objective was to elucidate the complex relationship between codon optimality and mRNA stability. We utilized LISCA to evaluate mRNA stability and expression rates of various exogenous eGFP-mRNA reporters versus CayRFP-mRNA reference systems. We observed a consistent decrease in mRNA lifetime for five mutated eGFP mRNA constructs containing slow-codon windows at specific sites compared with the original eGFP reporter. In the presence of targeting siRNAs, however, the knockdown effect was seemingly attenuated by the introduction of slow codons, resulting in overall stabilities comparable to unmutated eGFP mRNA.

A possible explanation of these findings is that suboptimal codons stabilize mRNA integrity against RNAi, mediated via modulation of the ribosome density profile. Alternatively, our results may indicate the interference of different mRNA degradation mechanisms when both slow-codon windows and siRNA are present. The regulation of both endogenous and exogenous mRNA stability is a complex process, influenced in part by the translation process itself [11,76]. As mRNA translation is a stochastic process, ribosome collisions can occur and trigger rescue mechanisms [38,47,48,50]. As previously noted in various studies, synonymous codon exchange within the ORF comes with a well-described adverse effect of codon suboptimality on mRNA lifetime [11,22,41,43,49]. In contrast, for *Saccharomyces cerevisiae*, a protective effect was reported for slow-codon windows that are located close to the 5′ end of the ORF and potentially decrease the density of ribosomes further downstream [77].

We confirmed the destabilizing effect of slow-codon windows on eGFP mRNA in two different human cell lines and found it to be dependent on the position of the slow-codon window, but not on the location of the siRNA-binding site. Based on simulated ribosome density profiles, we assume that the translational machinery is slowed down in a slow-codon window position-dependent manner. The introduced changes in ribosomal density may then mediate both the triggering of rescue mechanisms and a potential protection against siRNA-RISC attack. Moreover, we computed the correlation of predicted ribosomal densities and measured mRNA stability for all codon positions in the ORF, where the first part of the ORF appeared as a particularly sensitive region in both tested cell lines. A high predicted ribosome density within this region is positively correlated with low mRNA stability in general but has a seemingly protective effect against siRNA-mediated degradation. This observation was independent of the position of the siRNA-binding site. It may relate to the well-known ramp effect, which describes the role of slow translation within the first 30–50 codons of the ORF in avoiding ribosome jams, thereby optimizing translation efficiency [78,79]. Furthermore, we observed that the local modification of ribosome speed produced a similar effect: our model predicts a peak of ribosomal density at the position of the slow codons, followed by a decrease below the reference after the slow-codon window. This ramp-effect-like observation may reduce the interaction of RISC with the translational machinery, thereby increasing mRNA stability, as previously described [57]. Differences between the tested cell lines are expected, e.g. due to differences in gene expression and metabolic characteristics of both cell types. Additionally, it has been previously reported that microRNA (miRNA)–mRNA interactions can be cell-specific [80], which may also apply to the siRNA–mRNA interactions studied here. However, it remains an open question which mechanisms lead to the observed correlation profiles of ribosome density and mRNA stability under RNAi. RNAi-mediated degradation is initiated by binding of the siRNA seed region [67,81]. Therefore, we mapped potential seed-binding regions on the ORF for siRNA 1 and 2 to investigate if this explains the observed position dependence. Indeed, an overall increased frequency of putative siRNA seed-binding regions was identified towards the 5′-end (electronic supplementary material, figure S13). While the constructs were carefully designed to avoid the creation of additional full binding sites, we observed that the construct containing the slow-codon window at codon position 48 exhibited a loss of a putative full seed-binding region due to the codon exchange. This modification may contribute to increased stability in that specific case. However, for the other constructs, no seed-binding sites were affected. Thus, this finding cannot fully explain the observed stabilization effects. Nonetheless, we cannot rule out the possibility of long-range interactions, as recently described by Bader & Tuller [80]. Previous studies showed a close interplay between ribosome movement and RISC binding [82,83]. In principle, mRNA secondary structure could impact the effects described here [26,80]. Through the literature, secondary structure is discussed to affect RISC target accessibility as, for example, described by Ruijtenberg *et al*. [57], Brown *et al*. [84] or Ameres *et al*. [68]. A theoretical structure prediction using RNAfold [85] for the five eGFP constructs did not provide any hints towards this direction (electronic supplementary material, figure S14), although we cannot ultimately rule out that changes in mRNA stability under RNAi are caused by altered secondary structures. However, focusing on just the secondary structure of an mRNA neglects the potential role of ribosomes. Ruijtenberg *et al.* described how translating ribosomes de-mask RISC binding sites on the mRNA [57]. This mechanism can explain the reduced stability for the unmutated eGFP sequence but not the beneficial effect of the slow-codon windows. In summary, the ability to observe protein expression trajectories of hundreds of single cells in parallel by LISCA together with simulations of mRNA translation by our software OCTOPOS allows us to demonstrate that translation dynamics can be manipulated via synonymous codon choice to actively control and modulate mRNA stability. We uncovered that siRNA-mediated decay can mitigate the stability disadvantage of mRNAs containing non-optimal codon windows. This approach could be exploited in future applications of exogenous mRNAs, especially in potential co-delivery systems.

## 4. Material and methods

### 4.1. Cell culture

A549 cells (DSMZ, AC107) were cultured in RPMI medium (Roswell Park Memorial Institute 1640, Gibco^TM^, Thermo Fisher Scientific, no. 11875093) supplemented with 10% (v/v) fetal bovine serum (no. 10270106) at 37°C and 5% CO_2_. For HuH7 cell culture, additionally, 5 mM HEPES (Gibco^TM^, Thermo Fisher Scientific, no. 15630080) and 1 mM Na-Pyruvate (Gibco^TM^, Thermo Fisher Scientific, no. 11360070) were added. For live-cell imaging, A549 cells were cleaved with T/E (trypsin/EDTA; Gibco 15400-054), HuH7 cells with Acutase (Invitrogen, 00-4555-56) and seeded in growth medium at a cell density of 1 × 10^6^ cells ml^−1^ (A549) or 5 × 10^5^ cells ml^−1^ (HuH7) with 25 µl per channel of the six-channel µ-slide (ibidi, no. 80600). After 60 min, cells were washed with OptiMEM (Reduced Serum Minimal Essential Medium; Gibco^TM^, Thermo Fisher Scientific, no. 31985062), which was also the medium for any transfection experiment. Cell lines were tested for mycoplasma contamination before experiments and were found to be negative.

### 4.2. Single-cell array fabrication

For the preparation of the single-cell pattern, photo-induced CuSO_4_ click reaction was carried out to selectively bind the cell-adhesive cyclo-Arg-Gly-Asp (RGD) to a cell-repellent PVA surface. Therefore, each channel of a bioinert, PVA-coated, µ-slide (ibidi, no. 80600) was filled with 33 µl of a 5 mM Diazirin (Enamine) and 10% (v/v) DMSO (Thermo Fisher Scientific, no. D12345) solution. The slide is illuminated with an in-house UV-illumination lamp (Rapp, 365 nm). To enable selective illumination, a silica-wafer-based photomask with 20 × 20 µm squares and 85 µm spacer was used. Following a washing step, the click reaction solution was applied (10 mM BTTA (Jena Bioscience, CLK-067-25), 2 mM CuSO_4_ (Jena Bioscience, CLK-MI005), 0.1 mM cyclo-RGD-azide (Lumiprobe, A1330), 100 mM vitamin C (Jena Bioscience, CLK-MI005) in sodium-phosphate buffer (Jena Bioscience, no. CLK-073)) for 1 h at room temperature. Click mix was removed, and the slide was washed with phosphate-buffered saline (PBS, Biochrom GmbH no. L182) several times (see also electronic supplementary material, figure S15).

### 4.3. mRNA and siRNA

eGFP constructs were kindly provided by Ethris GmbH; sequences of the ORFs are provided in the electronic supplementary material. CayRFP mRNA was produced as described previously in [60]. mRNA was produced without modified nucleotides. siRNAs were purchased from Dharmacon (P-002048-01-20). Sequences are available in the electronic supplementary material.

### 4.4. Transfection assay

To investigate translation kinetics, cells were transfected with equal amounts of CayRFP-mRNA and the respective eGFP-mRNA. Therefore, mRNAs were mixed with Lipofectamine2000 (Thermo Fisher Scientific, no. 11668019) according to the manufacturer’s protocol. In total, 2 ng µl^−1^ mRNA concentration transfection mixed was applied, and cells were incubated under physiologic conditions for 45 min. Afterwards, cells were washed in OptiMEM, and 12.5 pmol of the respective siRNA was applied for 30 min. Then, cells were washed in L15 medium (Gibco^TM^, no. 21083027), supplemented with 10% (v/v) fetal calf serum (FCS).

### 4.5. Fluorescence microscopy

Time-resolved fluorescence images were recorded with a Nikon TI Eclipse microscope. To allow image acquisition over 30 h, cells were incubated with the Oku-lab incubation system (cage incubator with active humidity and temperature control) under physiologic conditions. Channels were scanned in a 10 min time interval with 10× magnification (Nikon Objective, MRH00101). Bright-field (BF) illumination was carried out with a 100 W warm white LED (MHLED100W) and fluorescence with an LED light source (Lumencor, SOLA-SE II). eGFP fluorescence was captured with the eGFP Filterset (Chroma, F46-002), and CayRFP fluorescence was captured with the DsRed ET Filterset (Chroma, F46-005). Images were captured with a CMOS camera (PCO, pco.edge4.2). Acquisition control was performed with the NIS-Elements Advanced Research software (Nikon).

### 4.6. Image processing and analysis

Images obtained from NIS software were converted into time-resolved ‘.tif’ stacks. With the in-house analysis software PyAM [63], cell segmentation, tracking and background correction based on Schwarzfischer [64] were performed. To improve the signal-to-noise ratio, integrated fluorescence over a square region of interest with a side length of 350% of the square side length was analysed.

### 4.7. Calibration for absolute protein numbers

Conversion of fluorescence intensity into protein numbers was carried out with a polydimethylsiloxane (PDMS) calibration chip with known channel dimensions and protein solutions in different concentrations as described previously [60].

### 4.8. Data fitting

A non-linear least square fit of data to a three-stage maturation model (see figure 1C) for mRNA translation was performed. This system is described with the following system of differential equations:


$$\frac{\partial mRNA\left(t\right)}{\partial t}=\;-\;\delta \times mRNA\left(t\right),$$

$$\frac{\partial eGFP^{*}\left(t\right)}{\partial t}=\;-\;\beta \times eGFP^{*}\left(t\right)+k_{tl}\times mRNA\left(t\right)-k_{m}\times eGFP^{*}\left(t\right),$$

$$\frac{\partial eGFP\left(t\right)}{\partial t}=\;-\;\beta \times eGFP\left(t\right)+k_{m}\times eGFP^{*}\left(t\right),$$

where mRNA(t) is the concentration of mRNA, ${eGFP}^{*}\left(t\right)$ is the concentration of nascent eGFP, eGFP(t) is the concentration of maturated eGFP, δ is the degradation rate of mRNA, β is the degradation rate of nascent or maturated eGFP, $k_{TL}$ is the translation rate of eGFP encoding mRNA and $k_{m}$ is the maturation rate of eGFP.

Independent measurement of *k*_*m*_ and *ß* according to Krzysztoń *et al.* [60] via induction of translational stop with cycloheximide allowed to reduce the set of free parameters and thus the determination of mRNA degradation and expression rate by model fitting.

### 4.9. Data normalization and error calculation

If not stated differently, each experiment was repeated at least three times independently.

All single-cell data presented here are normalized to the co-transfected reference mRNA. Therefore, for each single cell n, eGFP stability $\Gamma_{\;eGFP}$ was normalized to respective CayRFP stability $\Gamma_{CayRFP}$ of the same cell.


$$\tau_{n}=\;\frac{\Gamma_{GFP}}{\Gamma_{RFP}}\;and\;accordingly\;for\;the\;expression\;rates:\;k_{TL,n}=\;\frac{K_{TL,\;eGFP}\;}{K_{TL,\;CayRFP}}.$$

To compare stabilities or expression rates of mutated constructs with corresponding values of unmutated eGFP, data for all separate constructs *i* in each experimental condition *j* (w/o, siCtrl, siRNA 1, siRNA 2) were normalized to the median eGFP mRNA stability in the same experimental condition,$FC\tau_{n,i,j}=\frac{\tau_{n,i,j}}{{\tilde{\tau }}_{eGFP,j}}$. Those values are presented in the graphs unless stated otherwise. For simplicity, only the formula for stability is shown here. Accordingly, expression rates were normalized where applicable.

Figure 4D aimed to compare the RNAi effects only. Therefore, for every single cell n in siRNA *j* experiments (j=1,2), fold change $FC\tau_{n,i,j}$ of mRNA stability in the presence of siRNA *j* = 1 and *j* = 2 was normalized to the corresponding median fold change $FC{\tilde{\tau }}_{i,siCtrl}$ of mRNA stability in presence of siCtrl,


$$norm.\;FC\;\tau_{n,i,j}=\;\frac{FC\;\tau_{n,i,j}}{FC\;{\tilde{\tau }}_{i,\;siCtrl}}.$$

The *p*-values correspond to results from Mann–Whitney *U* tests and are indicated in the plots as assigned in the following: (****) for *p* < 10^–4^, (***) for *p* < 10^–3^, (**) for *p* < 10^–2^, (*) for *p* < 5 × 10^–2^ and (ns) for *p* > 5 × 10^–2^.

Correlation between measured stability and ribosome occupancy was calculated using the Pearson correlation coefficient.

## Data Availability

The single-cell data are available at Zenodo [86]. Codes for OCTOPOS [70] and PyAMA [63] were published previously. Supplementary material is available online [87].
